# An Evolutionary Game Theory Study for Construction and Demolition Waste Recycling Considering Green Development Performance under the Chinese Government’s Reward–Penalty Mechanism

**DOI:** 10.3390/ijerph17176303

**Published:** 2020-08-29

**Authors:** Hongyu Long, Hongyong Liu, Xingwei Li, Longjun Chen

**Affiliations:** 1 School of Civil Engineering and Geomatics, Southwest Petroleum University, Chengdu 610500, China; Liuhy@swpu.edu.cn; 2 College of Architecture and Urban-Rural Planning, Sichuan Agricultural University, Chengdu 611830, China; 3 School of Management, Jiangsu University, Zhenjiang 212013, China; 4 Institute for Advanced Materials and Technology, University of Science and Technology Beijing, Beijing 100083, China; chenlongjun530@126.com

**Keywords:** construction and demolition waste (CDW), supply chain management, green development performance (GDP), evolutionary game theory, construction industry

## Abstract

The low efficiency of the closed-loop supply chain in construction and demolition waste (CDW) recycling has restricted the green development of China’s construction industry. Additionally, the government’s reward–penalty mechanism has a huge influence on green development. This study aimed to investigate the effect of green development performance (GDP) and the government’s reward–penalty mechanism on the decision-making process of production and recycling units, as well as to reveal the optimal strategies under different conditions. Therefore, the strategies’ evolutionary paths of production and recycling units were investigated by using evolutionary game theory. Firstly, an evolutionary game model between production units and recycling units was proposed under the government’s reward–penalty mechanism. Then, the evolutionary stability strategies in different scenarios were discussed. Finally, the effects of the relevant parameters on the evolutionary paths of the game model were analyzed using numerical simulations. The main conclusions are as follows. (1) When the range of GDP changes, the evolutionary stable strategy changes accordingly. GDP plays a positive role in promoting the high-quality development of the CDW recycling supply chain, but an increase in GDP can easily lead to the simultaneous motivation of free-riding. (2) The government’s reward–penalty mechanism effectively regulates the decision-making process of production and recycling units. An increase in the subsidy rate and supervision probability helps to reduce free-riding behavior. Moreover, the incentive effect of the subsidy probability on recycling units is more obvious, while the effect of the supervision probability on improving the motivation of active participation for production units is more remarkable. This paper not only provides a decision-making basis to ensure production and recycling units to make optimal strategy choices under different conditions but also provides a reference for the government to formulate a reasonable reward–penalty mechanism that is conducive to a macro-control market.

## 1. Introduction

The concept of green development is widely accepted due to increasingly serious global environmental issues [1]. Green development performance (GDP) can evaluate the effect of green development behavior, including economic, environmental, and social performance [2]. Green building effectively promotes the green development of the construction industry, but it inevitably produces construction and demolition waste (CDW) [3]. One typical example is the recycling of CDW. Improving the recovery rate of CDW is beneficial to the economy, the environment, and society, so as to improve the GDP. Existing research shows that the recovery rate of CDW in China is relatively low and the critical reasons for this are the lack of supply of raw material and difficulties associated with selling remanufactured products in CDW recycling units [4]. Furthermore, due to the pursuit of profit maximization, there is a free-riding phenomenon in CDW recycling, which rationally drives people to make use of other people’s efforts to obtain profit [5]. However, free-riding has always been a problem for enterprises in terms of improving their overall performance [6]. The resulting effect is that it is difficult for the closed-loop supply chain to achieve high-quality development. The current recovery rate of CDW in China is 5%, while in advanced countries such as Japan and those in the European Union, it has exceeded 90% [7].

The decision-making process of stakeholders in the supply chain is key to promoting a better formation and operation of the closed-loop supply chain for CDW recycling in China [8]. At the same time, the government plays an important role in supply chain management [9]. By adjusting the subsidy rate and supervision probability, the government can control the distribution pattern of enterprise profits, thus affecting the decision-making behavior of stakeholders in the closed-loop supply chain [10,11]. Therefore, the government’s reward–penalty mechanism should be considered in the decision-making process of the closed-loop supply chain. Many scholars have studied the game behavior between stakeholders of CDW recycling in the closed-loop supply chain. Shen et al. [12] studied the decision-making behavior between production and recycling units on the basis of game theory and the prospect theory of behavioral economics. Lu and Huang [13] constructed game matrixes for production and recycling units within the government, analyzing their decision-making behavior. Both of these studies suggested that government subsidies can effectively promote the development of the CDW recycling supply chain. However, there are a limited number of studies that consider the effect of GDP and free-riding behavior. Free-riding behavior is not conducive to group development because it affects the initiative of individuals [14]. Therefore, free-riding behavior should be considered in the decision-making process of the closed-loop supply chain. In order to maintain the operation and development of an enterprise, the decision-maker is most concerned about profitability [15]. The social reputation and corporate image gained by production units in the process of participating in the closed-loop supply chain belong to social performance, which can promote the sustainable development of enterprises [16]. Accordingly, consumers’ green preference belongs to environmental and economic performance. Consumers’ green preference has a significant effect on their green purchase behavior, which affects the profit of recycling units [17]. Therefore, GDP should be considered in the decision-making process of the closed-loop supply chain.

For CDW recycling units, there is a lack of supply of raw material and it is difficult to sell remanufactured products in China. Therefore, this paper is the first to determine game players. Game strategies were determined by said players. The game players in the closed-loop supply chain are CDW production and recycling units. The production units sell CDW to the recycling units and the recycling units sell remanufactured goods to the production units, thus forming a closed-loop supply chain. Therefore, under the government’s reward–penalty mechanism, what kind of strategy should the production units adopt? Should they actively participate in the closed-loop supply chain and provide CDW to the recycling units (i.e., active or negative participation)? Furthermore, what kind of strategy should the recycling units adopt? Should they produce remanufactured products with a high quality (i.e., high-quality or non-high-quality remanufacturing)? These have become important decision-making problems in the closed-loop supply chain of CDW recycling, which comprise the game strategies of players in this paper.

Then, how does GDP and the government’s reward–penalty mechanism affect the strategic choices of production and recycling units? This study aimed to investigate the effect of GDP and the government’s reward–penalty mechanism on the decision-making process of production and recycling units, revealing the optimal strategies for these units under different circumstances. To the best of the authors’ knowledge, herein, for the first time, GDP was introduced into the evolutionary game model to study the optimal decision-making process of CDW recycling players (i.e., production and recycling units) under the Chinese government’s reward–penalty mechanism. On the one hand, this paper enriches the literature on evolutionary game theory and green development, and provides a theoretical basis for the decision-making process of CDW recycling in other countries or regions. On the other hand, this paper provides a decision-making basis for CDW production and recycling units to make optimal strategy choices under different conditions. It also provides a reference for the government to formulate a reasonable reward–penalty mechanism, which is conducive to a macro-control market.

The remainder of this paper is structured as follows: Section 2 provides a relevant literature review; Section 3 constructs a game model between production and recycling units under the government’s reward–penalty mechanism; Section 4 analyzes the stability of each equilibrium point and determines the evolutionary stability strategy (ESS) in different scenarios; Section 5 presents the numerical simulation and discusses the influence of relevant parameters on the game’s evolutionary path; finally, Section 6 summarizes the conclusions and limitations of this paper.

## 2. Literature Review

### 2.1. CDW Recycling in the Government’s Reward–Penalty Mechanism

At present, there are two main alternatives for disposing of CDW in China, i.e., landfills and recycling [18]. From a short-term perspective, the traditional method of landfilling is simple and cost-effective. However, CDW recycling, which is an alternative with a circular economy concept, is a feasible way of solving the increasing amount of CDW [19]. The initial investment capital for CDW recycling is relatively large, but it is still a very promising treatment process because of its many advantages. Due to the limited carrying capacity of landfills and the limited amount of natural materials, CDW recycling can effectively save land resources by easing the burden on landfills and can also protect non-renewable natural resources [20,21]. Moreover, CDW recycling is a circular economy model based on the “reduce, reuse, and recycle” concept, forming a closed-loop supply chain [22]. Through the development of a closed-loop supply chain, the contradiction between economic development and the ecological environment can be improved, so as to achieve green development [23]. The current practical results of CDW recycling in China are not satisfactory and one way to improve this condition is via the government’s reward–penalty mechanism, which can effectively regulate the decision-making behavior of production and recycling units [24]. (1) In terms of the reward mechanism, government subsidies can increase the green behavior motivation of enterprises and can quickly improve the performance of the recycling supply chain [25]. Government subsidies can increase the profit of production and recycling units, so as to encourage them to participate in the supply chain with high–quality [26]. However, the subsidy incentive may not be enough to offset the high cost, because the degree of benefit of the subsidized units depends on the financial capacity of the government. Therefore, the establishment of a reasonable reward mechanism has an important impact on the decision-making process of production and recycling units. (2) Regarding the penalty mechanism, in terms of production units, the fee charged by the government for production units to dispose of CDW can effectively control the amount of CDW generated. Lu and Tam [27] found that the implementation of a CDW disposal charging scheme is the most effective policy for reducing the pressure on CDW landfill sites. However, exorbitant disposal fees can increase the possibility of illegal dumping [28]. In terms of recycling units, the government can control the quality of remanufactured products produced by recycling units through a punishment mechanism. This is because the quality of remanufactured products can affect consumers’ purchase intentions, which can then affect the development of the CDW recycling supply chain [29]. Therefore, the establishment of an appropriate penalty mechanism has an important impact on the decision-making process of production and recycling units.

### 2.2. GDP

As is well known, GDP is an indicator of the effect of green development behavior [30]. Many scholars have conducted research in this field. For example, Wang et al. [31] believed that the comprehensive utilization of waste glass can promote the green development of building materials. Ebrahimi et al. [32] found that carbon dioxide can continuously convert CDW into construction cement. Mickovski et al. [33] used recycled building materials as green roofs to improve the sustainable benefits of buildings. The above research proves that the green development behavior of CDW recycling contributes to the green development of the construction industry. In the field of CDW recycling, traditional research mainly focuses on economic benefits [34]. From a broader perspective, economic, environmental, and social performance must be simultaneously considered in research [35]. The government plays an important role in GDP (i.e., economic, environmental, and social performance). (1) In terms of economic performance, government guidance can promote the green transformation of consumption, thereby increasing the consumer demand for green products, which ultimately benefits the economy of a recycling unit [36]. Improvement in the economic performance of a recycling unit can directly increase its profits and can result in an increased willingness to produce high-quality remanufactured products [37], which, in turn, can improve consumers’ purchase intentions. Therefore, through high-quality production to expand the market, recycling units can obtain higher profits. (2) In terms of environmental performance, the formulation of policies affects the driving force of the development of green products in enterprises, and ultimately benefits the environment [38]. As consumers are more willing to buy green products, improving the environmental performance of a recycling unit can indirectly increase its profits by expanding the market [39]. Therefore, improving of the environmental performance of a recycling unit results in a greater willingness of said unit to produce high-quality remanufactured products. (3) Regarding social performance, the innovation, initiative, and social value orientation of enterprises have a positive effect. The formulation of policies and regulations by the government can stimulate an enterprise’s initiative, and can ultimately improve their corporate social performance [40]. Furthermore, the active participation of production units in the supply chain can improve their social performance and can indirectly increase their profits.

It can be seen that GDP is closely related to the profits of production and recycling units, which means that GDP can affect the decision-making behavior of these units by influencing their profits [41]. Therefore, the effect of GDP on the profits of stakeholders in the CDW recycling supply chain is evident and the government can thus promote the improvement of GDP. However, limited research has focused on the effect of GDP on the decision-making process of production and recycling units, especially in the evolutionary game model. Therefore, this paper investigates the effect of GDP on the decision-making behavior of production and recycling units under the government’s reward–penalty mechanism in order to be able to thoroughly explain the effect of GDP on the evolutionary path of the game player’s strategic choices.

### 2.3. Application of Evolutionary Game Theory

Evolutionary game theory is a theoretical method that determines how bounded rational players make decisions under the background of incomplete information [42]. This method emphasizes the dynamic equilibrium of the system. Many scholars now use evolutionary game theory to solve problems in various fields, including economics [43], computer science [44], and management [45]. Evolutionary game theory is also widely used to solve supply chain management issues. Babu and Mohan [46] used evolutionary game theory to explain and analyze the social and economic sustainability of the public health insurance supply chain. Naini and Jafarieskandari [47] combined evolutionary game theory and the environmental supply chain to propose a mixed performance evaluation system to evaluate a company’s sustainable management. Evolutionary game theory can analyze the effect of each parameter on the decision-making behavior of players in the game model, revealing the evolutionary path of their strategic choices. Therefore, evolutionary game theory can be used as a research method to study the effect of GDP on the decision-making process of game players.

Many researchers have applied evolutionary game theory to CDW recycling issues. The government wants production units to recycle CDW to reduce environmental pollution [48], yet production units are usually reluctant to do so because recycling is more complex and expensive than landfilling. Therefore, the government needs to provide subsidies as a means for production units to increase their profits. However, subsidies for production units will increase the government’s expenditure, which may lead to their hesitation in implementing a subsidy incentive [49]. In this sense, a conflict in interest between the government and production units has emerged. Chen et al. [50] studied the evolutionary game behavior between the government and production units, determining appropriate subsidies and fines. Therefore, evolutionary game theory is suitable for use when studying the conflicts of interest in the process of CDW recycling. As the study of CDW recycling becomes more in-depth, scholars have considered more comprehensive stakeholders. On the basis of the government and production units, Su [51] introduced recycling units into the game model, studying the effect of government supervision and policies on the decision-making process of stakeholders in CDW recycling. He and Yuan [52] also considered the effect of consumer quality perception and put forward corresponding policy suggestions. Therefore, evolutionary game theory is an effective method for studying multi-party conflicts of interest and for making policy recommendations. In this paper, the potential GDP brought from consumers and society is considered in the game model. In this way, the game environment can be restored more comprehensively and accurately, and conclusions can be drawn more accurately. In addition, different from classical game theory, evolutionary game players constantly observe and imitate each other in the process of interaction, so as to optimize the strategy [53]. Furthermore, the difference in GDP and the government’s reward–penalty mechanism can lead to different strategies. At present, limited research considers free-riding behavior when studying multiple stakeholders. Understanding the strategy changes of production and recycling units can help to reduce free-riding behavior. Moreover, CDW recycling is a complex system problem. Therefore, the dynamic evolutionary process between production and recycling units is of great significance to understanding the role of GDP and the government’s reward–penalty mechanism.

## 3. Model Formulation

The game players in this study were CDW production and recycling units, both of which are stakeholders in the CDW recycling supply chain. Additionally, the game players were regarded as the decision-makers with bounded rationality. In the game environment in which the government encourages the market to develop a CDW recycling industry, this work studied the decision-making behavior of the stakeholders of the CDW recycling supply chain under the government’s reward–penalty mechanism, specifically whether the production unit actively participates in the supply chain and whether the recycling unit produces high-quality remanufactured products. Production and recycling units have different preferences for different strategies. With the evolution of GDP and the government’s reward–penalty mechanism, the two game players adjust their strategies by comparing the profits. Through continuous trial and error and learning, both players finally determine the most appropriate strategies.

To study the problem, the following assumptions were made. Table 1 provides definitions of the parameters involved in the assumptions.

**Assumption** **1.** To promote the high-quality development of the supply chain, this study assumes that all stakeholders are involved in the supply chain. The CDW generated by CDW activities is treated in different ways, according to its value: High-value CDW (e.g., metal and wood) is recycled directly, while CDW that cannot be directly recycled (e.g., waste concrete, masonry, etc.) is transported to landfills or is recycled into remanufactured products. The CDW studied in this paper is of the latter type with recycling value.

**Assumption** **2.** The production unit has two strategies, including active (AP) and passive (NP) participation in the supply chain. The recycling unit also has two strategies, including high-quality remanufacturing (H) and non-high-quality remanufacturing (NH). When the production unit chooses NP, the recycling unit chooses NH, and the profits of the production and recycling units are π_p_ and π_r_, respectively.

**Assumption** **3.** When the production unit chooses AP or the recycling unit chooses H, it increases their respective GDP. GDP is related to the economic level, which can improve the profits of enterprises [54]. In this study, the GDP of the production unit (k) is expressed in the form of the growth coefficient of the social reputation and corporate image for said production unit. Moreover, the GDP of the recycling unit (m) is expressed in the form of a coefficient of consumers’ green preferences. Moreover, the goal of high-quality development will increase the economic cost of technology, personnel, and other investments [55]. As for costs, the basic cost of the production unit (C_P_) is the CDW disposal cost. Active participation in the supply chain will incur additional costs (C_P_’), including human, finance, material, and other costs. The basic cost of the recycling unit (C_r_) is the cost of producing remanufactured products. High-quality remanufactured products will incur additional costs (C_r_’), including the costs of introducing advanced equipment, technology, and personnel.

**Assumption** **4.** The government will subsidize enterprises that promote the high-quality development of the supply chain. Setting different subsidy rates according to the degree of positivity of the enterprise helps to promote the efficient development of the industry [56]. When both of the game players choose a high-quality participation strategy, that is, the production unit chooses AP and the recycling unit chooses H, the subsidy rate for both players is λ. If only one player chooses a high-quality participation strategy, i.e., the production unit chooses AP or the recycling unit chooses H, the subsidy rate for the player is λ’(λ > λ’). To standardize the CDW recycling industry, the government will supervise the behavior of enterprises and impose fines for non-standard behavior. The probability of government supervision is α, and the fines for the production and recycling units are F_p_ and F_r_, respectively.

**Assumption** **5.** When the recycling unit chooses H and the production unit chooses NP, the production unit obtains profit from free-riding (Δπ_p_) with an increase in the market demand. Similarly, when the production unit chooses P and the recycling unit chooses NH, the recycling unit also obtains profit from free-riding (Δπ_r_).

**Assumption** **6.** It is supposed that the proportion of the production unit that chooses AP is x (0 ≤ x ≤ 1) and that of P is 1-x. In addition, it is assumed that the proportion of the recycling unit that chooses H is y (0 ≤ y ≤ 1), and that of NH is 1-y.

**Figure 1:**
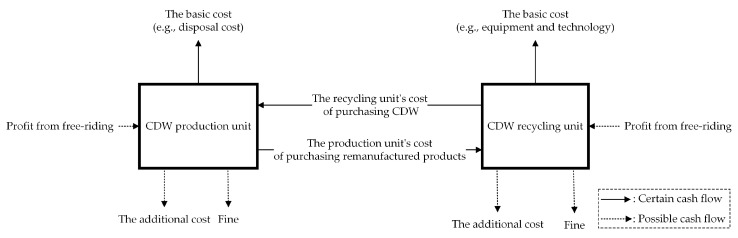
The cash flows for the construction and demolition waste (CDW) supply chain model.

The formation of the CDW recycling closed-loop supply chain is as follows. First, the production unit sells CDW to the recycling unit. Then, the recycling unit transforms the purchased CDW into remanufactured products. Finally, the recycling unit sells the remanufactured products to the production unit. Therefore, the supply chain model of CDW recycling can be proposed from the perspective of supply chain management, as can be seen in Figure 1. Based on the above six assumptions, the payoff matrix between the CDW production and recycling units was established under different strategies and is presented in Table 2.

## 4. Evolutionary Game Model Analysis

### 4.1. Calculation of Stable Points

According to Table 2, the expected payoffs of the CDW production unit for the strategies of AP and NP are as follows:

(1) $$U_{\mathrm{AP}}{\;=\;y[(1\;+\;m)\pi }_{p}{\;-\;C}^{\prime }{}_{p}{\;+\;\lambda (C}_{p}{\;+\;C}^{\prime }{}_{p}{)]\;+\;(1\;-\;y)[(1\;+\;m)\pi }_{p}{\;-\;C}^{\prime }{}_{p}{\;+\;\lambda^{\prime }(C}_{p}{\;+\;C}^{\prime }{}_{p})]$$

(2) $$U_{\mathrm{NP}}{\;=\;y(\pi }_{p}{\;-\;\alpha F}_{p}{\;+\;\Delta \pi }_{p}{)\;+\;(1\;-\;y)(\pi }_{p}{\;-\;\alpha F}_{p})$$

The expected payoffs of the CDW recycling unit for the strategies of H and NH are as follows:

(3) $$U_{H}\;=\;x[\left(1\;+\;k\right)\pi_{r}{\;-\;C}^{\prime }{}_{r}{\;+\;\lambda (C}_{r}{\;+\;C}^{\prime }{}_{r})]\;+\;(1\;-\;x)[\left(1\;+\;k\right)\pi_{r}\;-{\;C}^{\prime }{}_{r}{\;+\;\lambda^{\prime }(C}_{r}{\;+\;C}^{\prime }{}_{r})]$$

(4) $$U_{\mathrm{NH}}{\;=\;x(\pi }_{r}{\;-\;\alpha F}_{r\;}{+\;\Delta \pi }_{r}{)\;+\;(1\;-\;y)(\pi }_{r}{\;-\;\alpha F}_{r})$$

Replicator dynamics equations can describe the evolution of game players’ strategy over time. According to the asymmetric replicator dynamics equations proposed by Taylor and Jonker in 1978 [57], the replicator dynamics equation of the CDW production unit for the strategy of AP and that of the CDW recycling unit for the strategy of H are as follows:

(5) $$F\;=\;\frac{\mathrm{dx}}{\mathrm{dt}}\;=\;x\left(1\;-\;x\right){(U}_{\mathrm{AP}}{\;-\;U}_{\mathrm{NP}})\;=\;x\left(1\;-\;x\right)\{{m\pi }_{p}{\;-\;C}^{\prime }{}_{p\;}{+\;\alpha F}_{p}{\;+\;\lambda^{\prime }(C}_{p}{\;+\;C}^{\prime }{}_{p}{)\;+\;y[\lambda (C}_{p}{\;+\;C}^{\prime }{}_{p})\;-{\;\lambda^{\prime }(C}_{p}{\;+\;C}^{\prime }{}_{p}{)\;-\;\Delta \pi }_{p}]\}$$

(6) $$G\;=\;\frac{\mathrm{dy}}{\mathrm{dt}}\;=\;y\left(1\;-\;y\right){(U}_{H}{\;-\;U}_{\mathrm{NH}})\;=\;y\left(1\;-\;y\right)\{{k\pi }_{r}{\;-\;C}^{\prime }{}_{r}{\;+\;\alpha F}_{r}{\;+\;\lambda^{\prime }(C}_{r}{\;+\;C}^{\prime }{}_{r}{)\;+\;x[\lambda (C}_{r}{\;+\;C}^{\prime }{}_{r}){\;-\;\lambda^{\prime }(C}_{r}{\;+\;C}^{\prime }{}_{r}{)\;-\;\Delta \pi }_{r}]\}$$

According to the stability theory of first-order differential equations, let dx/dt = 0 and dy/dt = 0, the stable points of the system composed of Formulas (5) and (6) can be obtained, i.e., (0, 0), (0, 1), (1, 0), and (1, 1). Let $m_{0}\;=\;\frac{C^{\prime }{}_{p}{\;-\;\alpha F}_{p}{\;-\;\lambda^{\prime }(C}_{p}{\;+\;C}^{\prime }{}_{p})}{\pi_{p}}$, $m_{1}\;=\;\frac{C^{\prime }{}_{p}{\;-\;\alpha F}_{p}{\;-\;\lambda (C}_{p}{\;+\;C}^{\prime }{}_{p}{)\;+\;\Delta \pi }_{p}}{\pi_{p}}$, $k_{0}\;=\;\frac{C^{\prime }{}_{r}{\;-\;\alpha F}_{r}{\;-\;\lambda^{\prime }(C}_{r}{\;+\;C}^{\prime }{}_{r})}{\pi_{r}}$, $k_{1}\;=\;\frac{C^{\prime }{}_{r}{\;-\;\alpha F}_{r}{\;-\;\lambda (C}_{r}{\;+\;C}^{\prime }{}_{r}{)\;+\;\Delta \pi }_{r}}{\pi_{r}}$, $m_{0}<\;m_{1}$ and $k_{0}<\;k_{1}$, when $\left({\lambda \;-\;\lambda^{\prime })(C}_{r}{\;+\;C}^{\prime }{}_{r}\right){<\;\Delta \pi }_{r}$, $m_{0}<\;m\;<\;m_{1}$, and $k_{0}<\;k\;<\;k_{1}$, ${(x}^{\;*}{,\;y\;}^{*})\;=\;(\frac{C^{\prime }{}_{r}{\;-\;k\pi }_{r}{\;-\;\alpha F}_{r}{\;-\;\lambda^{\prime }(C}_{r}{\;+\;C}^{\prime }{}_{r})}{\left({\lambda \;-\;\lambda \prime )(C}_{r}{\;+\;C}^{\prime }{}_{r}\right){\;-\;\Delta \pi }_{r}},\frac{C^{\prime }{}_{p}{\;-\;m\pi }_{p}{\;-\;\alpha F}_{p}{\;-\;\lambda^{\prime }(C}_{p}\;+\;C^{\prime }{}_{p})}{\left({\lambda \;-\;\lambda^{\prime })(C}_{p}{\;+\;C}^{\prime }{}_{p}\right){\;-\Delta \pi }_{p}})$ is also one of the stable points.

### 4.2. Evolutionary Equilibrium Stability Analysis

The stable point obtained from the replicator dynamics equation is not necessarily an ESS and needs to be further calculated according to the method proposed by Friedman [58]. Through the local stability analysis of the Jacobian matrix of the system, an ESS can be obtained. The Jacobian matrix J of this system is:

(7) $$J\;=\left(\begin{array}{cc} \frac{\partial F}{\partial x} & \frac{\partial F}{\partial y}\\ \frac{\partial G}{\partial x} & \frac{\partial G}{\partial y} \end{array}\right)=\left(\begin{array}{cc} a_{11} & a_{12}\\ a_{21} & a_{22} \end{array}\right)$$

where

(8) $$a_{11}\;=\;(1\;-\;2x)\left\{{m\pi }_{p}{\;-\;C}^{\prime }{}_{p}{\;+\;\alpha F}_{p}{\;+\;\lambda^{\prime }(C}_{p}{\;+\;C}^{\prime }{}_{p}{)\;+\;y[\lambda (C}_{p}{\;+\;C}^{\prime }{}_{p}{)\;-\;\lambda^{\prime }(C}_{p\;}{+\;C}^{\prime }{}_{p}{)\;-\;\Delta \pi }_{p}]\right\}$$

(9) $$a_{12}{\;=\;x(1\;-\;x)[\lambda (C}_{p}{\;+\;C}^{\prime }{}_{p}){\;-\;\lambda^{\prime }(C}_{p}{\;+\;C}^{\prime }{}_{p}{)\;-\;\Delta \pi }_{p}]$$

(10) $$a_{21\;}{=\;y(1\;-\;y)[\lambda (C}_{r}{\;+\;C}^{\prime }{}_{r}){\;-\;\lambda^{\prime }(C}_{r}{\;+\;C}^{\prime }{}_{r}{)\;-\;\Delta \pi }_{r}]$$

(11) $$a_{22}\;=\;(1\;-\;2y)\left\{{k\pi }_{r}{\;-\;C}^{\prime }{}_{r}{\;+\;\alpha F}_{r}{\;+\;\lambda^{\prime }(C}_{r}{\;+\;C}^{\prime }{}_{r}{)\;+\;x[\lambda (C}_{r}{\;+\;C}^{\prime }{}_{r}){\;-\;\lambda^{\prime }(C}_{r}{\;+\;C}^{\prime }{}_{r}{)\;-\;\Delta \pi }_{r}]\right\}$$

A stable point is judged as an EES if it satisfies the following conditions: (1)$\;{\det\;(J)\;=\;a}_{11}a_{22}{\;-\;a}_{12}a_{21\;}>\;0$; (2)$\;{\mathrm{tr}\;(J)\;=\;a}_{11}{+\;a}_{22}\;<\;0$. Table 3 shows the values of a_11_, a_12_, a_21_, and a_22_ for each stable point.

Table 3 shows that tr (J) is equal to 0 at the stable point of (x*, y*), which does not satisfy the condition that tr (J) < 0 for the EES, so it is not an ESS of this system. Next, the stability of the remaining four stable points, namely, (0, 0), (0, 1), (1, 0), and (1, 1), is discussed:

(1) When 0 < m < m_0_ and 0 < k < k_0_, the ESS of this system is (0, 0).
(2) When 0 < m < m_0_ and k_0_ < k < 1, or m_0_ < m < m_1_ and k_1_ < k < 1, the ESS of this system is (0, 1).
(3) When m_0_ < m < 1 and 0 < k < k_0_, or m_1_ < m < 1 and k_0_ < k < k_1_, the ESS of this system is (1, 0).
(4) When m_0_ < m < m_1_ and k_0_ < k < k_1_, the ESS of this system is (0, 1) or (1, 0).
(5) When m_1_ < m < 1 and k_1_ < k < 1, the ESS of this system is (1, 1).

According to Table 3, the det (J) and tr (J) values of each stable point can be calculated, so the stability of each stable point under the above five cases can be judged. The results are shown in Table 4, Table 5, Table 6, Table 7 and Table 8.

The evolutionary paths of the strategies of the production and recycling units in the five cases can be obtained from Table 4 to Table 8, as follows.

In case (1), when 0 < m < m_0_ and 0 < k < k_0_, the ESS of this system is (0, 0). In this case, GDP is slight. No matter which strategy the recycling unit chooses, the profit of the production unit brought about by its GDP is lower than the cost of green development. For the recycling unit, no matter which strategy is chosen, the profits of the recycling unit brought about by its GDP are also lower than the cost of green development. Therefore, the production and recycling units ignore the risk of being fined by the government and tend to participate in the supply chain with relatively low quality. This case is common among production and recycling units with relatively poor performance in reality.

In case (2), when 0 < m < m_0_ and k_0_ < k < 1, or m_0_ < m < m_1_ and k_1_ < k < 1, the ESS of this system is (0, 1). In this case, the GDP of the recycling unit is improved, and the production unit can obtain profit from free-riding. For the production unit, the cost of green development is still greater than the sum of profits from its GDP and free-riding, so the production unit will choose passive participation. For the recycling unit, it obtains no profit from free-riding. However, the profits brought about by its GDP are large enough to balance out the cost, so the recycling unit will choose high-quality remanufacturing. This case is common among recycling units with good performance in reality.

In case (3), when m_0_ < m < 1 and 0 < k < k_0_, or m_1_ < m < 1 and k_0_ < k < k_1_, the ESS of this system is (1, 0). In this case, the GDP of the production unit is improved, and the recycling unit can obtain profits from free-riding. For the recycling unit, the cost of green development is still greater than the sum of profits from its GDP and free-riding, so the recycling unit will choose non-high-quality remanufacturing. For the production unit, it obtains no profit from free-riding. However, the profits brought about by its GDP are large enough to balance out the cost, so the production unit will choose active participation. This case is common among production units with good performance in reality.

In case (4), when m_0_ < m < m_1_ and k_0_ < k < k_1_, the ESS of this system is (0, 1) or (1, 0). In this case, the two players with medium performance form a very tight match. Besides, the cost of green development is less than the profits brought about by GDP, so both players want to choose free-riding to obtain more profits. Therefore, when the production unit chooses to actively participate, the recycling unit will choose non-high-quality remanufacturing. Accordingly, when the recycling unit chooses high-quality remanufacturing, the production unit will choose passive participation.

In case (5), when m_1_ < m < 1 and k_1_ < k < 1, the ESS of this system is (1, 1). In this case, the GDP of both players is relatively high. The profits brought about by GDP are large enough, so both players choose to participate in the supply chain with high-quality. This case is common among production and recycling units with good performance in reality.

### 4.3. Evolutionary Equilibrium Stability Analysis in Case (4) by Parameter Variation

**Figure 2:**
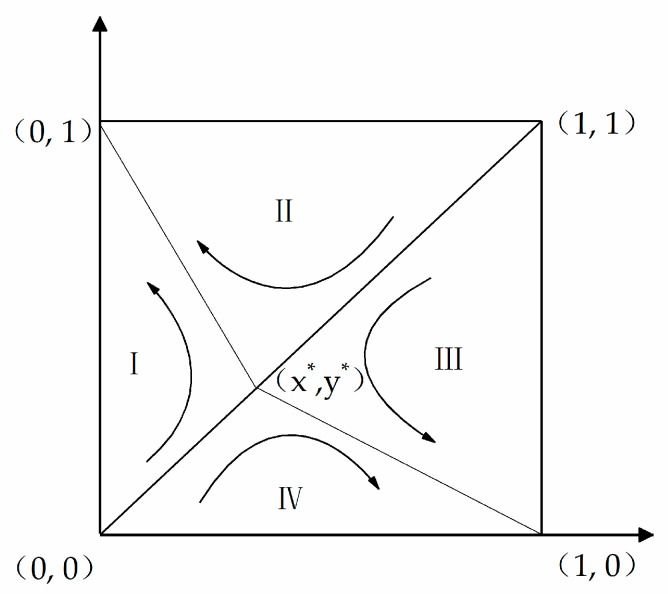
Phase diagram of the evolutionary game in case (4).

Game players with medium performance are more common, so this paper selected case (4) for further analysis, that is, the influence of parameters on strategy evolution when m_0_ < m < m_1_ and k_0_ < k < k_1_. Figure 2 shows a phase diagram of the evolutionary game in case (4).

Figure 2 shows that the square area is divided into four parts: I, II, III, and IV. It is assumed that S_1_ is the sum of areas I and II, and S_2_ is the sum of areas III and IV. The proportion of S_1_ to total strategy space relies on the initial value of each parameter in the game model. In view of the fact that the Chinese CDW recycling supply chain cannot be of a high quality, the domain is weighted toward the initial conditions (0, 0). The probability that the production and recycling units finally choose the strategies of (0, 1) and (1, 0) is determined by the proportion of S_1_ to S_2_ in the total square area.

(12) $$\begin{array}{l} S_{1}\;=\;\frac{1}{2}{\;\times \;1\;\times \;x}^{*}\;+\;\frac{1}{2}\;\times \;1\;\times \;\left({1\;-\;y}^{*}\right)\;=\;\\ \frac{1}{2}\left(\frac{C^{\prime }{}_{r}{\;-\;k\pi }_{r}{\;-\;\alpha F}_{r}{\;-\;\lambda^{\prime }(C}_{r}{\;+\;C}^{\prime }{}_{r})}{\left({\lambda \;-\;\lambda \prime )(C}_{r}{\;+\;C}^{\prime }{}_{r}\right){\;-\;\Delta \pi }_{r}}+\frac{{\lambda (C}_{p}{\;+\;C}^{\prime }{}_{p}{)\;-\;\Delta \pi }_{p}{\;-C}^{\prime }{}_{p}{\;+\;m\pi }_{p}{\;+\;\alpha F}_{p}}{\left({\lambda \;-\;\lambda \prime )(C}_{p}{\;+\;C}^{\prime }{}_{p}\right){\;-\;\Delta \pi }_{p}}\right) \end{array}$$

According to Formula (12), there are 15 parameters that influence the evolution of the system, i.e., m, k, α, λ, λ’, C_r_, C_r_’, C_p_, C_p_’, F_r_, F_p_, π_r_, π_p_, Δπ_r_, and Δπ_p_. As this paper primarily focused the effect of GDP and the government’s reward–penalty mechanism on the decision-making process of production and recycling units, the correlation of the relevant parameters was judged by partial derivatives. The results are shown in Table 9.

According to Table 9, when m_0_ < m < m_1_, k_0_ < k < k_1_, and the other parameters remain the same, with an increase in GDP for the production unit or a decrease in GDP for the recycling unit, the probability that the ESS is (1, 0) increases. On the contrary, with a decrease in GDP for the production unit or an increase in GDP for the recycling unit, the probability that the ESS is (0, 1) increases. The effect of the subsidy rate for both players with high-quality participation (λ) and government supervision probability (α) on the system is not clear, but the increase in the subsidy rate for only one existing player with high-quality participation (λ’) can increase the probability of the ESS being (0, 1).

## 5. Numerical Simulations and Discussion

Theoretically, the ideal ESS is that the production unit chooses the active participation strategy and the recycling unit chooses high-quality remanufacturing, that is, strategy (1, 1). However, according to the analysis of the evolutionary game model in Section 4, due to the different performance of each enterprise, the optimal strategy of the player in different scenarios is not always (1, 1). Thus, how can we make the production unit participate in the supply chain more actively? Additionally, how can we make the recycling unit pay more attention to the high-quality production of remanufactured products? This requires the consideration of the strength of enterprises and the reasonable cooperation of the government’s reward–penalty mechanism, such as subsidies and supervision, so as to realize the high-quality development of the CDW recycling supply chain.

A simulation and analysis of the evolutionary game model were conducted by MATLAB R2108a to intuitively discuss the effect of different parameters on the decision-making behavior of production and recycling units. Through an investigation of the literature [59,60], this study assumed the initial values of the parameters for λ, λ’, C_P_, C_r_, C_P_’, and C_r_’. Through the expert consultation method, the initial values of the parameters were assumed, i.e., α, π_p_, π_r_, Δπ_p_, and Δπ_r_. The ranges of m and k in case (4) were 0.0006 < m < 0.466 and 0.0843 < k < 0.3229, respectively, from which the initial values of m and k were determined. Table 10 shows the parameter values.

### 5.1. The Effect of GDP on the Game Equilibrium

**Figure 3:**
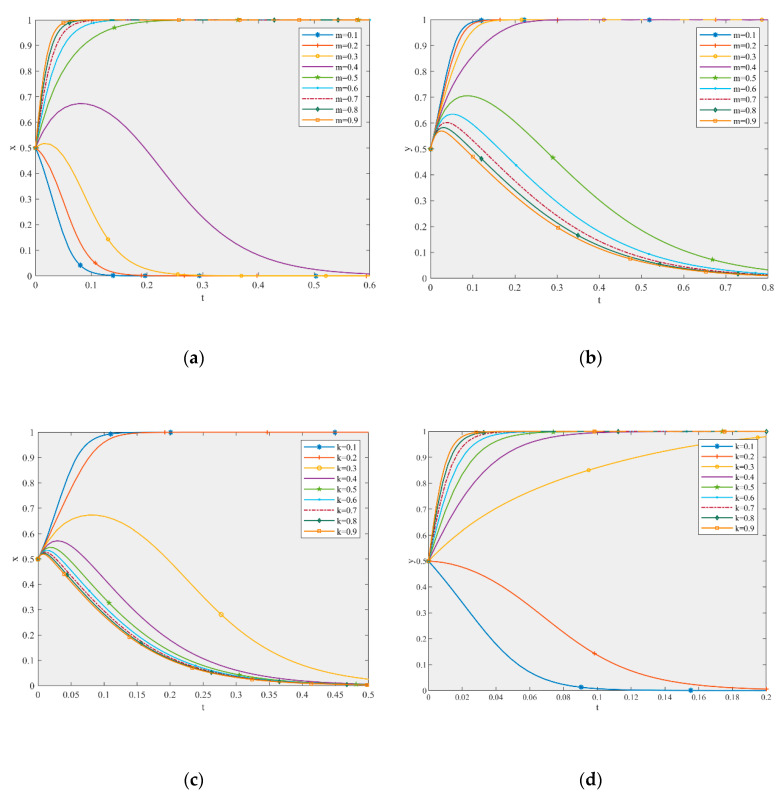
The effect of m (**a**) on the production unit and (**b**) the recycling unit, and the effect of k (**c**) on the production unit and (**d**) the recycling unit in the game equilibrium.

According to the stability analysis of the stable point in Section 4.2, the ESS of the production and recycling units will change with different GDPs. Based on the parameter settings, this paper simulated the influence of parameters m and k on the game equilibrium, as shown in Figure 3.

Figure 3a,b shows that when the other parameters remain unchanged and the value of m changes from 0.1 to 0.9, the ESS of the game model changes from (0, 1) to (1, 0), which is consistent with the analysis results of m in Table 9. Compared to the five curves converging to 1 in Figure 3a, the larger the value of m, the faster it converges to the stable state of active participation. Moreover, when the value of m is 0.5, the profit of the production unit is 199.92. Additionally, when the value of m is 0.9, the profit is 255.92. Thus, the change in profit is obvious. However, with an increase in m, the convergence rate of non-high-quality remanufacturing is faster. In other words, an increase in GDP for the production unit will make said unit more likely to actively participate in the supply chain. The greater the GDP, the faster the system converges to (AP, NH), but this will lead to free-riding behavior of the recycling unit.

Figure 3c,d shows that when the other parameters remain unchanged and the value of k changes from 0.1 to 0.9, the ESS of the game model changes from (1, 0) to (0, 1), which is consistent with the analysis results of k in Table 9. Compared to the seven curves converging to 1 in Figure 3d, the larger the value of k, the faster it converges to the stable state of high-quality remanufacturing. Additionally, when the value of k is 0.3, the profit of the recycling unit is 330.4, and when the value of k is 0.9, the profit is 498.4. Thus, the change of profit is obvious, and the promoting effect is greater than that of m. However, with an increase in k, the convergence speed of the passive participation of production units also becomes faster. In other words, an increase in GDP for the recycling unit will make said unit more likely to choose high-quality remanufacturing. The greater the GDP, the faster the system converges to (NP, H), but this will lead to free-riding behavior of the production unit.

Consequently, GDP plays a positive role in promoting the high-quality development of the CDW recycling supply chain [61]. The greater the GDP, the more obvious the promoting effect, and the GDP of recycling units plays a more important role. At the same time, an increase in GDP easily encourages motivation for free-riding. When the performances of two companies are similar, they should pay attention to one another’s free-riding motivation and choose carefully.

### 5.2. The Effect of the Government’s reward–Penalty Mechanism on the Game Equilibrium

#### 5.2.1. The Effect of Parameters λ and λ’

**Figure 4:**
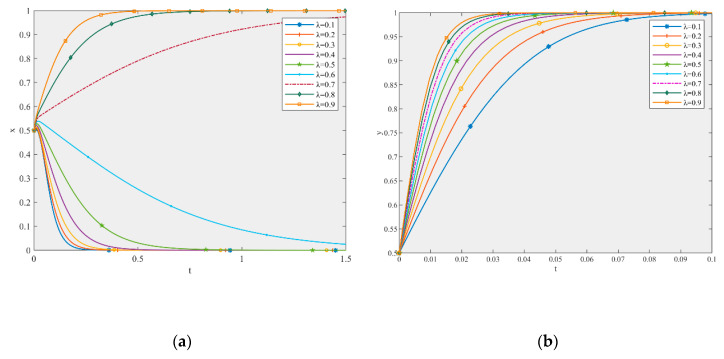
The effect of λ (**a**) on the production unit and (**b**) the recycling unit in the game equilibrium.

**Figure 5:**
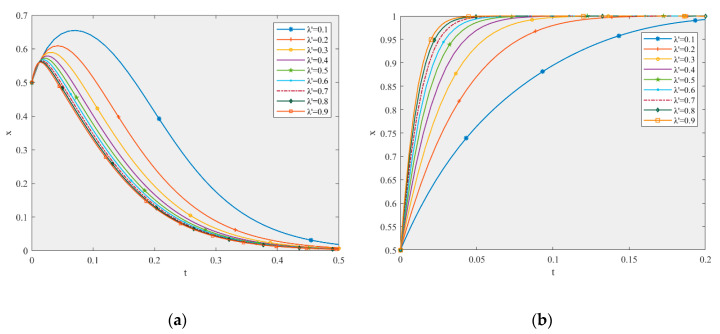
The effect of λ’ (**a**) on the production unit and (**b**) the recycling unit in the game equilibrium.

Based on the parameter settings, this study simulated the influence of parameters λ and λ’ on the game equilibrium shown in Figure 4 and Figure 5.

According to Figure 4, under the initial values of the parameters, the ESS of the production and recycling units is (0, l). When the other parameters are constant, with an increase in the value of λ, the ESS changes to (1, l). As Figure 4a shows, when λ changes from 0.1 to 0.9, the strategy of the production unit changes from negative to active participation. The larger the λ, the faster the system converges to the stable state of active participation. When λ increases from 0.7 to 0.9, the profit of the production unit increases from 202.3 to 212.1. Therefore, the profit changes obviously. Figure 4b shows that, for the recycling unit, no matter how much λ increases, the strategy is always high-quality remanufacturing. From the perspective of the convergence time, the time it takes for the recycling unit to reach the stable state is very short (i.e., 0.1 s). Therefore, the promoting effect of λ on the recycling unit is very obvious and an increase in λ can effectively shorten the time required for the recycling unit to converge to the stable state of high-quality remanufacturing. Moreover, with an increase in λ, the strategy of the production unit changes to active participation, but this does not mean that the recycling unit has motivation to free-ride. Therefore, a government subsidy can discourage free-riding behavior.

According to Figure 5, under the initial values of the parameters, the ESS of the production and recycling units is (0, l). When the other parameters are constant, with an increase in λ’, the ESS remains unchanged, but the system converges to the stable state faster. Therefore, an increase in λ’ can only accelerate the convergence rate of the system, and has little effect on the strategic choice of the production and recycling units.

Consequently, government subsidies play a positive role in promoting the high-quality development of the CDW recycling supply chain and the promoting effect on the recycling unit is more obvious than that on the production unit. The greater the subsidy rate, the more obvious the promoting effect. This is because government subsidies can directly reduce costs, while government subsidies are limited and can only reduce costs to a certain extent. Enterprises can not only rely on subsidies to reduce costs, but should establish a better collaborative relationship with cooperative enterprises to create a new supply chain management mode of CDW recycling [62]. Moreover, government subsidies can effectively control the probability of free-riding. This is contrary to the conclusions of some studies, which stated that subsidies may increase the occurrence of free-riding [63,64]. This may be due to the fact that their formulation of subsidies is different from that presented in this paper. Both strategies available to the subsidized person can be subsidized to a certain extent in their paper. However, the subsidy set in this paper can only be obtained by high-quality participation in the game. Therefore, the government should pay attention to the formulation of subsidy rates for the high-quality participation of both players and that of only one existing player, especially for the former one, so as to reduce the phenomenon of free-riding and to increase the motivation of the production and recycling units to participate with high-quality.

#### 5.2.2. The Effect of Parameter α

**Figure 6:**
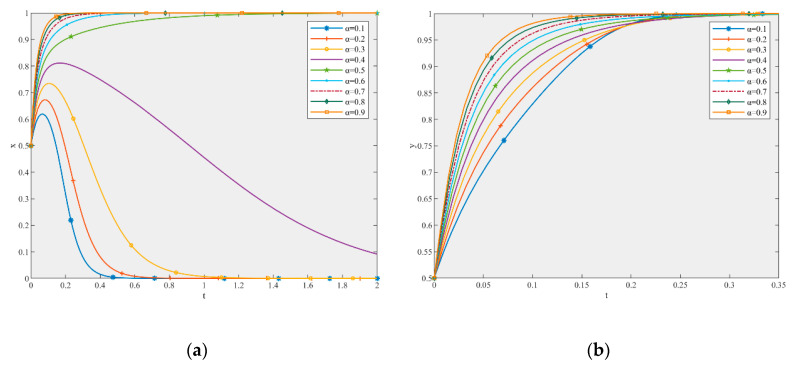
The effect of α (**a**) on the production unit and (**b**) the recycling unit in the game equilibrium.

Based on the parameter settings, this paper simulated the influence of parameter α on the game equilibrium in Figure 6.

According to Figure 6, under the initial values of the parameters, the ESS of the production and recycling units is (0, l). When the other parameters are constant, with an increase in α, the ESS becomes (1, 1). As Figure 6a shows, when α changes from 0.1 to 0.9, the strategy of the production unit changes from negative to active participation. The larger the α, the faster the system converges to the stable state of active participation. Figure 6b shows that, no matter how much α increases, the strategy choice of the recycling unit is always high-quality remanufacturing. In terms of the convergence time, it takes more than 2 s for the production unit to reach the stable state, while only 0.35 s is required for the recycling unit. The speed at which the recycling unit reaches a stable state is much faster than that of the production unit. Moreover, an increase in α can effectively shorten the time required for the recycling unit to converge to the stable state of high-quality remanufacturing. With an increase in α, the strategy of the production unit changes to active participation, but this does not mean that the recycling unit has motivation to free-ride, indicating that compared to the recycling unit, the government should strengthen the supervision of the production unit. Moreover, government regulation can discourage free-riding behavior.

Consequently, government supervision plays a positive role in promoting the high-quality development of the CDW recycling supply chain. The higher the supervision probability, the more obvious the promoting effect. The improvement of the government supervision probability can not only improve the enthusiasm of the production unit to participate actively, but can also effectively control the probability of free-riding behavior [65,66]. Therefore, public policies such as specific regulations and mandatory degrees of normative standards should be used to strengthen the supervision of production and recycling units [67], especially production units, so as to reduce the phenomenon of free-riding and to improve the enthusiasm for the high-quality participation of production and recycling units.

## 6. Conclusions 

In this paper, evolutionary game theory was used to study the optimal decision-making process of CDW recycling considering GDP under the government’s reward–penalty mechanism. The optimal strategies of production and recycling units in different scenarios were also discussed and the following conclusions were drawn: (1) GDP, government subsidies, fines, benefits from free-riding, and costs affect the decision-making process of production and recycling units; (2) GDP in different value ranges leads to changes in the ESS, as GDP plays a positive role in promoting the high-quality development of the CDW recycling supply chain, but an increase in GDP can easily lead to motivation to free-ride; (3) the government plays an important role in promoting the high-quality development of the CDW recycling supply chain. The government’s reward–penalty mechanism effectively regulates the decision-making process of production and recycling units. An increase in the subsidy rate and supervision probability helps to reduce free-riding behavior. The incentive effect of the subsidy rate on recycling units is more obvious, while the effect of the supervision probability on improving the motivation for active participation of production units is more obvious.

### Implications

Based on the above conclusions, the following management implications can be drawn. (1) For production and recycling units, they should pay more attention to the improvement of the internal factor of GDP. When the GDP of an enterprise is good enough, enterprises should be cautious about the free-riding motivation of cooperative enterprises and should try to choose cooperative enterprises with a strong sense of social responsibility. (2) For the government, the formulation of a reasonable reward–penalty mechanism can not only encourage the high-quality participation of production and recycling units in the supply chain, but can also reduce free-riding behavior. The government should pay attention to the formulation of subsidy rates for the high-quality participation of both players and that of only one existing player, especially for the former one. Moreover, public policies such as specific regulations and mandatory degrees of normative standards should be used to strengthen the supervision of production and recycling units, with that of recycling units being less important. According to the GDP level of the production and recycling units in the market, a reasonable subsidy rate and supervision probability can be determined through the evolutionary game model, which makes it possible for the government to macro-control the market.

There are some limitations to this paper. Based on the correlation analysis of the parameters in this paper, the correlation of the supervision probability and the subsidy rate for the high-quality participation of both players is not clear enough. Additionally, there may be inflection points or peaks between them, which need to be further clarified in future research.

## Figures and Tables

**Table 1:** Definition of parameters.

Unit	Parameter	Definition
Government	λ	The subsidy rate when both players participate in the game with high quality
	λ’	The subsidy rate when only one player participates in the game with high quality
	α	Probability of government supervision
CDW production unit	m	Green development performance
	π_p_	Profit from passive participation
	Δπ_p_	Profit from free-riding
	C_P_	Basic cost
	C_P_’	Additional cost
	F_p_	Fine
CDW recycling unit	k	Green development performance
	π_r_	Profit from non-high-quality production of remanufactured products
	Δπ_r_	Profit from free-riding
	C_r_	Basic cost
	C_r_’	Additional cost
	F_r_	Fine

Note: CDW (construction and demolition waste).

**Table 2:** The payoff matrix between a production unit and a recycling unit.

		CDW Recycling Unit	
		H(y)	NH(1−y)
CDW production unit	AP (x)	${(1+m)\pi }_{p}-C^{\prime }{}_{p}{+\lambda (C}_{p}{+C}^{\prime }{}_{p})$	${(1+m)\pi }_{p}-C^{\prime }{}_{p}{+\lambda^{\prime }(C}_{p}{+C}^{\prime }{}_{p})$
		$\left(1+k\right)\pi_{r}-C^{\prime }{}_{r}{+\lambda (C}_{r}{+C}^{\prime }{}_{r})$	$\pi_{r}-{\alpha F}_{r}{+\Delta \pi }_{r}$
	NP (1−x)	$\pi_{p}-{\alpha F}_{p}{+\Delta \pi }_{p}$	$\pi_{p}-{\alpha F}_{p}$
		$\left(1+k\right)\pi_{r}-C^{\prime }{}_{r}{+\lambda^{\prime }(C}_{r}{+C}^{\prime }{}_{r})$	$\pi_{r}-{\alpha F}_{r}$

**Table 3:** The values of a_11_, a_12_, a_21_, and a_22_ for each stable point.

Stable Point	a_11_	a_12_	a_21_	a_22_
(0, 0)	${m\pi }_{p}-C^{\prime }{}_{p}{+\alpha F}_{p}{+\lambda^{\prime }(C}_{p}{+C}^{\prime }{}_{p})$	0	0	${k\pi }_{r}-C^{\prime }{}_{r}{+\alpha F}_{r}{+\lambda^{\prime }(C}_{r}{+C}^{\prime }{}_{r})$
(0, 1)	${m\pi }_{p}-C^{\prime }{}_{p}{+\alpha F}_{p}{+\lambda (C}_{p}{+C}^{\prime }{}_{p}{)-\Delta \pi }_{p}$	0	0	${-[k\pi }_{r}-C^{\prime }{}_{r}{+\alpha F}_{r}{+\lambda^{\prime }(C}_{r}{+C}^{\prime }{}_{r})]$
(1, 0)	${-[m\pi }_{p}-C^{\prime }{}_{p}{+\alpha F}_{p}{+\lambda^{\prime }(C}_{p}{+C}^{\prime }{}_{p})]$	0	0	${k\pi }_{r}-C^{\prime }{}_{r}{+\alpha F}_{r}{+\lambda (C}_{r}{+C}^{\prime }{}_{r}{)-\Delta \pi }_{r}$
(1, 1)	${-[m\pi }_{p}-C^{\prime }{}_{p}{+\alpha F}_{p}{+\lambda (C}_{p}{+C}^{\prime }{}_{p}{)-\Delta \pi }_{p}]$	0	0	${-[k\pi }_{r}-C^{\prime }{}_{r}{+\alpha F}_{r}{+\lambda (C}_{r}{+C}^{\prime }{}_{r}{)-\Delta \pi }_{r}]$
(x*, y*)	0	−	−	0

Note: because the values of a_12_ and a_21_ for (x*, y*) are not related to the analysis, they are not calculated.

**Table 4:** Stability analysis of the points in case (1).

	Det(J)	Tr(J)	Stability
(0, 0)	+	−	ESS
(0, 1)	−	?	Saddle point
(1, 0)	−	?	Saddle point
(1, 1)	+	+	Unstable point

Note: “+” indicates that the calculation result is greater than zero, “−” is less than zero, and “?” is uncertain. ESS (evolutionary stability strategy).

**Table 5:** Stability analysis of the points in case (2).

Point	0 < m < m_0_, k_0_ < k < 1			m_0_ < m < m_1_, k_1_ < k < 1		
	Det(J)	Tr(J)	Stability	Det(J)	Tr(J)	Stability
(0, 0)	−	?	Saddle point	+	+	Unstable point
(0, 1)	+	−	ESS	+	−	ESS
(1, 0)	+	+	Unstable point	−	?	Saddle point
	−	?	Saddle point			
(1, 1)	+	+	Unstable point	−	?	Saddle point
	−	?	Saddle point			

Note: “+” indicates that the calculation result is greater than zero, “−” is less than zero, and “?” is uncertain. ESS (evolutionary stability strategy).

**Table 6:** Stability analysis of the points in case (3).

Point	m_0_ < m < 1, 0 < k < k_0_			m_1_ < m <1, k_0_ < k <k_1_		
	Det(J)	Tr(J)	Stability	Det(J)	Tr(J)	Stability
(0, 0)	−	?	Saddle point	+	+	Unstable point
(1, 0)	+	+	Unstable point	−	?	Saddle point
	−	?	Saddle point			
(0, 1)	+	−	ESS	+	−	ESS
(1, 1)	−	?	Saddle point	−	?	Saddle point
	+	+	Unstable point			

Note: “+” indicates that the calculation result is greater than zero, “−” is less than zero, and “?” is uncertain. ESS (evolutionary stability strategy).

**Table 7:** Stability analysis of the points in case (4).

Point	Det(J)	Tr(J)	Stability
(0, 0)	+	+	Unstable point
(0, 1)	+	−	ESS
(1, 0)	+	−	ESS
(1, 1)	+	+	Unstable point
(x*, y*)	+	0	Central point

Note: “+” indicates that the calculation result is greater than zero, “−” is less than zero, and “?” is uncertain. ESS (evolutionary stability strategy).

**Table 8:** Stability analysis of the points in case (5).

Point	Det(J)	Tr(J)	Stability
(0, 0)	+	+	Unstable point
(0, 1)	−	?	Saddle point
(1, 0)	−	?	Saddle point
(1, 1)	+	−	ESS

Note: “+” indicates that the calculation result is greater than zero, “−” is less than zero, and “?” is uncertain. ESS (evolutionary stability strategy).

**Table 9:** Correlation analysis of the parameters in the system.

**Parameter**	**m**	**k**	**α**	**λ**	**λ’**
	↑	↑	↑	↑	↑
**S_1_**	↓	↑	U	U	↑

Note: U indicates that the correlation of the parameters is uncertain.

**Table 10:** The parameter values.

Parameter	Value	Parameter	Value
(x_0_, y_0_)	(0.5, 0.5)	π_p_	140
m	0.3	π_r_	280
k	0.4	Δπ_p_	70
α	0.2	Δπ_r_	80
λ	0.08	C_P_	35
λ’	0.12	C_r_	270
F_p_	50	C_P_’	14
F_r_	50	C_r_’	60
